# Cryosurgery Versus Primary Androgen Deprivation Therapy for Locally Recurrent Prostate Cancer After Primary Radiotherapy: A Propensity-Matched Survival Analysis

**DOI:** 10.7759/cureus.7983

**Published:** 2020-05-06

**Authors:** Glenn Bauman, Keyue Ding, Joseph Chin, Shiva Nair, Alessandra Iaboni, Juanita Crook, Laurence Klotz, David Dearnaley, Eric Horwitz, Christopher O'Callaghan

**Affiliations:** 1 Radiation Oncology, London Regional Cancer Program - London Health Sciences Centre, London, CAN; 2 Canadian Cancer Trials Group, Queen's University, Kingston, CAN; 3 Surgery - Division of Urology, Western University, London, CAN; 4 Surgery - Division of Urology, London Health Sciences Centre, London, CAN; 5 Canadian Clinical Trials Group, Queen's University, Kingston, CAN; 6 Radiation Oncology, BC Cancer Agency, Kelowna, CAN; 7 Urology, University of Toronto, Toronto, CAN; 8 Radiation Oncology, Royal Marsden United Kingdom Trust, London, GBR; 9 Radiation Oncology, Fox Chase Cancer Center, Philadelphia, USA; 10 Canadian Clincial Trials Group, Queen's University, Kingston, CAN

**Keywords:** prostate cancer, recurrent, radiation, salvage

## Abstract

Background

Optimal management of isolated local recurrence of prostate cancer after primary radiotherapy remains to be defined. Up-front androgen deprivation therapy (ADT) is widely used but may adversely affect the quality of life and is essentially a palliative treatment. Local salvage carries a different side-effect profile and is potentially curative, but it has not been compared to ADT.

Materials and methods

We conducted a propensity-matched analysis of cohorts of men treated with either whole gland cryotherapy (CRYO) or primary ADT following the diagnosis of locally recurrent prostate cancer. Our specific objectives were to compare overall survival (OS) and prostate cancer-specific mortality (PCSM) between CRYO vs. ADT.

Results

After a one-to-one matching, 169 patients from each cohort were included in comparisons. Median follow-up time was 6.7 years (ADT) vs. 18 years (CRYO). The 10-year PCSM was 18.5% (ADT) vs. 16.2% (CRYO), which was not statistically different [hazard ratioo (HR): 0.69, 95% CI: 0.36-1.34, p=0.27]. The median OS was 12.3 years (CRYO) versus 10.2 years (ADT) (HR: 0.63, 95% CI: 0.42-0.95, p=0.03).

Conclusions

While PCSM was similar between the two strategies, CRYO was associated with a longer OS compared to primary ADT. Given the retrospective nature of the trial, these results should be considered hypothesis-generating, and phase III trials comparing these two options are required to further explore these findings.

## Introduction

Optimal management of radio-recurrent prostate cancer remains undefined. While some patients may do well with expectant management, others may benefit from active treatment due to patient preference or concerns about aggressive biology. For active salvage treatment, both salvage androgen deprivation therapy (ADT) and local salvage therapies have been described [1-4]. There is a gap in the literature due to a lack of comparisons between up-front systemic and local salvage therapies. Literature-based comparisons such as systematic reviews suffer from selection bias inherent in choosing between these two modalities whereby ADT might be favored in men with suspected metastatic disease or those who are unfit for local salvage with ablative salvage therapy, which is offered to fitter men with suspected locally recurrent disease [5]. To address this knowledge gap, and by acknowledging the potential selection bias inherent in such comparisons, we undertook a propensity-matched analysis of local vs. up-front systemic post-radiotherapy salvage using two existing cohorts: an institutional series of whole gland cryotherapy (CRYO) and the prospective, randomized PR7 trial (NCT00003653) of intermittent vs. continuous ADT (both arms pooled as ADT for this analysis as the trial demonstrated no significant difference between these schedules) [1,6,7]. Our specific objectives were to compare overall survival (OS) and prostate cancer-specific mortality (PCSM) between CRYO vs. ADT.

## Materials and methods

CRYO cohort

Outcomes for the CRYO cohort have been previously reported [7]. All CRYO patients were treated between 1994 and 2004 and had histologic confirmation of local recurrence and negative restaging studies with CT and bone scan prior to treatment. Patients may have received ADT prior to treatment, but no patients received routine ADT post CRYO. ADT post CRYO was started on the basis of physician discretion in the setting of clinical, biochemical, or histologic evidence of recurrence.

ADT cohort

The Canadian Cancer Clinical Trial Group (CCTG) PR-7 trial enrolled 1,386 men between 1999 and 2005 in a comparison between intermittent and continuous salvage ADT for men with biochemical failure [rising prostate-specific antigen (PSA) of >3.0] after primary radiation or radiation after prostatectomy [1]. All patients had negative restaging with CT and bone scan prior and were at least one year from prior radiotherapy or ADT (if given with primary radiotherapy). As the outcomes for the men who received intermittent or continuous hormone therapy were not statistically different, we combined the continuous and intermittent arms in this prospective randomized trial in generating the propensity-matched ADT cohort for comparison to CRYO.

Inclusion of participants

Initially, CRYO contained 187 patients and ADT contained 1,386 patients. Patients were excluded from the analysis if they did not have primary radiotherapy as their initial treatment or had missing information for any of the baseline factors necessary for the propensity scoring analysis. Specifically, within CRYO, 15 (8.0%) were excluded because of incomplete histologic grading and biochemical or outcome data, leaving a total of 172 patients. Within ADT, 159 (11.5%) were excluded because they had prior prostatectomy prior to radiotherapy, 105 (7.5%) because of missing Gleason Score data, and three (0.2%) because of missing data regarding time from original radiotherapy, leaving a total of 1,119 patients.

Baseline factors for propensity-matched analysis

Based on the literature review on important factors of the disease population and the available data from both data resources, five factors were pre-selected for the propensity scoring analysis: Gleason Score at diagnosis (<7 vs. 7 vs. >7); time since completion of primary radiotherapy (one to three vs. >3 years); the history of prior neoadjuvant or adjuvant ADT (yes vs. no); PSA level at the time of salvage therapy (<4 vs. 4-10 vs. >10 ng/ml); age at the time of salvage therapy (≤70 vs. >70 years old). A comparison of the distribution of these factors by cohort is listed in Table 1.

**Table 1:** Distribution of propensity-matched variables between cohorts after matching ADT: androgen deprivation therapy; CRYO: cryotherapy; RT: radiotherapy; PSA: prostate-specific antigen

Variable	ADT (n = 169)	CRYO (n = 169)
Gleason grade <7	93	100
Gleason grade 7	55	57
Gleason grade >7	21	21
Time since primary RT <3 years	30	23
Time since primary RT >3 years	139	146
PSA at salvage <4	16	19
PSA at salvage 4-10	104	121
PSA at salvage >10	49	29
Age at salvage <70 years	67	77
Age at salvage >70 years	102	92

Outcomes of interest

PCSM was defined as death due to prostate cancer in both populations. Patients who remained alive or who died of non-cancer or undetermined causes were censored at their time of last follow-up/death. OS was defined as the time from initial salvage therapy until death from any cause (as an event) or last follow-up (as a censoring). As was conducted for the progression-free survival outcome, the patient from the CRYO population with all outcome data missing was censored on the day after their date of salvage treatment. In order to determine if the use of ADT prior to CRYO had an effect on these endpoints, we pre-specified subgroup analyses of CRYO patients who did or did not receive ADT prior to CRYO propensity-matched against the ADT population.

Propensity scoring analysis

One-to-one matching was undertaken to select individual ADT matches for the CRYO population based on propensity scoring analysis conducted using a logistic regression model with all baseline covariates included to predict treatment with either CRYO or ADT [6]. Individual matching was completed using an algorithm based on an allowable absolute difference in the propensity probability of each individual while maximizing the number of individuals who could be matched. The allowable width for matching was selected to be the smallest possible value in each situation, which resulted in the complete generation of at least one match for all CRYO patients. To account for residual variation within a stratum, raw linear propensity scores were included in the outcomes analysis.

## Results

**Figure 1:**
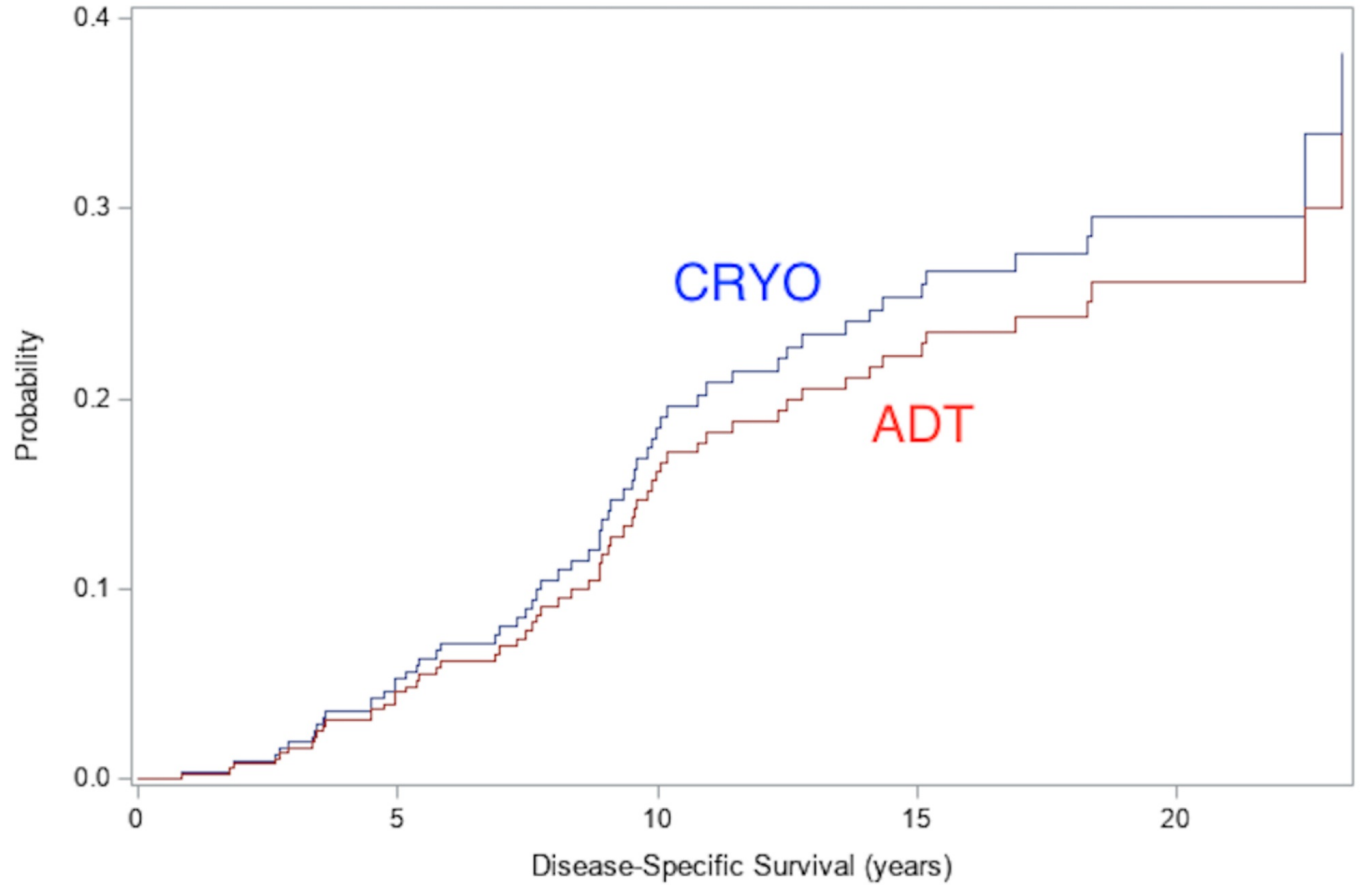
Prostate cancer-specific mortality: cumulative incidence curve comparing deaths from prostate cancer among CRYO vs. ADT cohort ADT: androgen deprivation therapy; CRYO: cryotherapy

For the matched cohorts, the median follow-up time was 6.73 years (95% CI: 6.23-6.93) among ADT and 18.65 years (95% CI: 17.95-19.90) among CRYO. Competing risk analysis was used to compare the PCSM between CRYO and ADT. The cumulative incidence function by treatment groups is displayed in Figure 1. The 10-year risk of prostate cancer death was 18.5% for patients in PR7, while it was 16.2% for patients in the CRYO cohort. Overall, there was no difference in PCSM between ADT and CRYO (HR: 0.76; 95% CI: 0.38-1.48, p=0.41; Fine-Gray regression model). When raw linear propensity scores were also included in the model to account for additional variability within propensity score strata, the adjusted PCSM HR for CRYO was 0.69 (95% CI: 0.36-1.34, p=0.27; FIgure 1).

**Figure 2:**
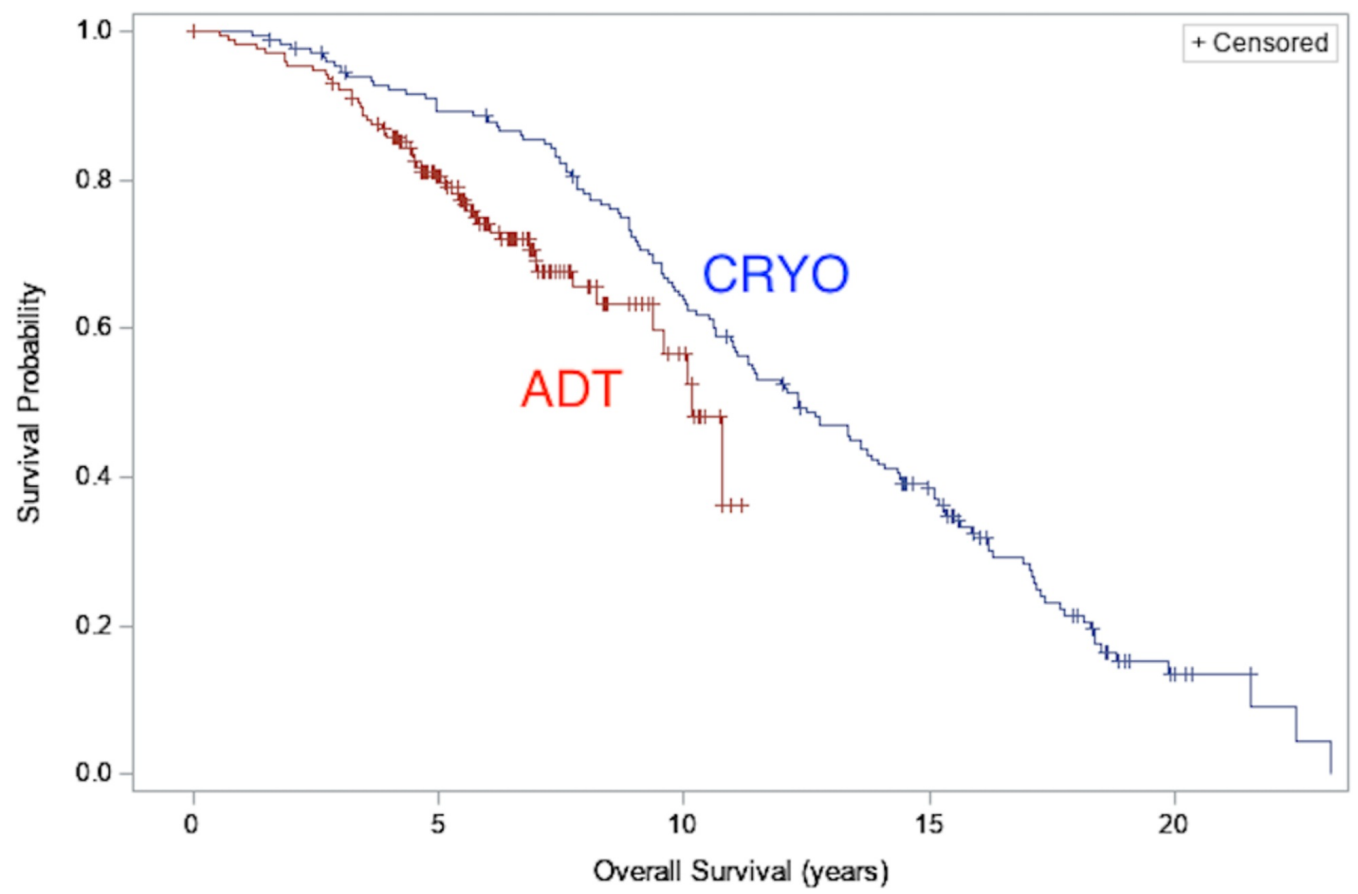
Overall survival: comparison of overall survival between CRYO and ADT cohorts ADT: androgen deprivation therapy; CRYO: cryotherapy

The median OS time for the CRYO population was 12.33 years (95% CI: 11.02-13.84) versus 10.17 years (95% CI: 9.38-N/A) for ADT (HR: 0.69, 95% CI: 0.45-1.06, log-rank p=0.086; Figure 2). When raw linear propensity scores were also included in the proportional hazard model to account for additional variability within propensity score strata, the adjusted HR still favored CRYO (HR: 0.63, 95% CI: 0.42-0.95, p=0.03; Figure 2).

OS and PCSM results were examined using propensity matching among the pre-defined subsets of men who did or did not receive ADT prior to salvage CRYO. Results for these subgroups were no different from those for the full CRYO population. The median time to ADT institution (for the CRYO cohort) was approximately 11 years, and the actuarial rate of ADT institution was 90% by 20 years post CRYO. In comparison, the time to develop hormone resistance was approximately seven years, and the actuarial rate of castrate resistance was approximately 70% at 10 years.

## Discussion

The optimal management of radio-recurrent prostate cancer remains undefined. While some men may do well with expectant management (lower PSA, longer doubling time), others may require active treatment due to either patient preference or concerns about more aggressive biology (higher PSA or shorter PSA doubling time) [8]. ADT is a widely accepted salvage strategy and carries the advantage of addressing both potential local and distant recurrence of prostate cancer. Randomized studies have demonstrated the equivalence of continuous and intermittent ADT approaches [1]. However, concerns about the routine use of ADT remain as the quality of life may be adversely affected even with intermittent approaches, and medical complications such as osteoporosis and metabolic syndrome may be triggered [2]. Finally, some men with isolated local recurrence may be forgoing potentially curative local salvage for an essentially palliative approach as ADT resistance inevitably develops over time.

Local salvage therapies offer a chance to ablate locally recurrent cancer while avoiding or deferring ADT use. A variety of local salvage therapies exist, including salvage prostatectomy, ablative modalities such as CRYO, high-intensity focused ultrasound, interstitial laser, and repeat radiotherapy such as brachytherapy (or, more recently, stereotactic external-beam approaches) [3,4]. In our CRYO cohort, the majority of men eventually required ADT, suggesting that most men had subclinical metastatic disease in addition to local failure. That said, the median time to ADT institution was over 10 years, thus avoiding potential toxicity of ADT for a substantial period.

Despite their availability, these local salvage modalities are infrequently deployed because of concerns about potential morbidity, variability in access to the expertise and technology for these specialized salvage therapies, and concerns about futile treatment in the setting of subclinical metastatic as well as local recurrence. As advanced imaging techniques for prostate cancer become more common [i.e. prostate-specific membrane antigen-positron emission tomography (PSMA-PET), multi-parametric magnetic resonance imaging (mpMRI)] and with the emergence of new therapeutic approaches (improvements in ablative therapies and surgical techniques), there is renewed interest in local therapies with deferred ADT treatment [9-11].

While there are no randomized trials to compare local salvage modalities, systematic reviews suggest similar efficacy (roughly 50% long-term biochemical control) with rates of significant toxicity (albeit with differing side-effect profiles) [3,4]. Recent propensity-matched analyses of institutional series suggest comparable efficacy between modalities such as CRYO and salvage prostatectomy [12]. However, no such comparisons exist between up-front salvage ADT and local salvage. To address this information gap for men being considered for salvage therapy, we conducted a propensity-matched analysis between an institutional cohort of men treated with salvage CRYO and men who proceeded directly to ADT on the randomized PR7 trial [1,7]. We found that CRYO was associated with a longer OS but similar PCSM compared to up-front ADT. These findings may be a consequence of differing lengths of follow-up, differences in patient co-morbidities not controlled for in propensity analysis, or longer exposure to adverse ADT effects on bone, cardiovascular, metabolic, and cognitive health among the ADT cohort [2,13].

Our findings have some limitations. For example, important endpoints such as toxicity and quality of life were not captured, and given the potential morbidity associated with both systemic and local salvage options, future comparisons incorporating these aspects would be valuable. We were also limited in the number of variables that were common between the two databases in terms of propensity matching. In particular, we had no variable reflective of the relative “fitness” of the two groups, which likely introduced an OS bias in favor of the CRYO group, similar to bias noted in other retrospective comparisons of prostate cancer therapies [5]. As with most reports of local salvage interventions, our CRYO group was limited to a single institution where the necessary expertise had been developed, and our findings may not be generalizable to other ablative salvage therapies.

## Conclusions

Prostate ablation (in this case with whole gland CRYO) was associated with an improved OS but not PCSM compared to salvage ADT in radio-recurrent prostate cancer. Given the retrospective nature of our findings, these results should be viewed as hypothesis-generating, and a randomized control trial comparing these options is necessary to examine potential prostate cancer-specific and OS differences as well as to examine other important endpoints such as quality of life and toxicity. Such a trial may need to adopt a pragmatic design where participating institutions randomize between "local salvage therapy of choice" and salvage ADT, given the lack of standardized local salvage approaches and the highly specialized nature of salvage ablative interventions.
